# Supplementary material for: MetaRibo-Seq measures translation in microbiomes
Source: Nat Commun. 2020 Jun 29;11:3268. doi: 10.1038/s41467-020-17081-z (PMC7324362; doi:10.1038/s41467-020-17081-z)
Supplement: Supplementary file 10 — Supplementary Data 7 [file 41467_2020_17081_MOESM10_ESM.zip › File2/Confidence_VeryHigh_Taxonomy/213621_out.krona.html]

Javascript must be enabled to view this page.

members
magnitude
magnitudeUnassigned
count
unassigned
taxon
rank

213621\_out

6

2
superkingdom
6

6
phylum
976

200643
6
class

171549
6
order

family
2
1853231

574697
genus
1

species
1
544645

SRS1041118\_contig\_number\_11311

283168

SRS097920\_contig\_number\_17550
1
genus

family
3
171552

838
3
genus

species
3
470565

SRS075765\_contig\_number\_21450SRS1041114\_contig\_number\_7009SRS142896\_contig\_number\_8007

815
family
1

816

SRS103987\_contig\_number\_1213
1
genus
